# CCN concentrations and BC warming influenced by maritime ship emitted aerosol plumes over southern Bay of Bengal

**DOI:** 10.1038/srep30416

**Published:** 2016-08-02

**Authors:** M. V. Ramana, Archana Devi

**Affiliations:** 1 Indian Institute of Space Science and Technology, Thiruvananthapuram, Kerala, 695 547, India

## Abstract

Significant quantities of carbon soot aerosols are emitted into pristine parts of the atmosphere by marine shipping. Soot impacts the radiative balance of the Earth-atmosphere system by absorbing solar-terrestrial radiation and modifies the microphysical properties of clouds. Here we examined the impact of black carbon (BC) on net warming during monsoon season over southern Bay-of-Bengal, using surface and satellite measurements of aerosol plumes from shipping. Shipping plumes had enhanced the BC concentrations by a factor of four around the shipping lane and exerted a strong positive influence on net warming. Compiling all the data, we show that BC atmospheric heating rates for relatively-clean and polluted-shipping corridor locations to be 0.06 and 0.16 K/day respectively within the surface layer. Emissions from maritime ships had directly heated the lower troposphere by two-and-half times and created a gradient of around 0.1 K/day on either side of the shipping corridor. Furthermore, we show that ship emitted aerosol plumes were responsible for increase in the concentration of cloud condensation nuclei (CCN) by an order of magnitude that of clean air. The effects seen here may have significant impact on the monsoonal activity over Bay-of-Bengal and implications for climate change mitigation strategies.

Marine vessels play an important role in the transport sector of the economy. However, seagoing ships pollute the clean marine environment and contribute significantly to the total anthropogenic emissions from the transportation sector. Transportation sector^1^ had contributed 14% of global greenhouse gas emission in 2010; to which international and coastal shipping had contributed 9%. The emissions from shipping are injected into the atmosphere in the form of coherent plumes, often at relatively high concentrations in relation to the atmospheric background concentrations.

Satellite measurements have confirmed the existence of high nitrogen dioxide (NO_2_) concentrations along the international shipping route in the Bay of Bengal^2^ (see Supplementary Fig. S1). Seasonal mean tropospheric columnar NO_2_ amounts over Bay of Bengal (BoB) for 2012 are shown in Fig. 1(a,b). The large expanse of NO_2_ values observed near the east coast of India is due to the continental outflow of pollution determined by the regional wind flow pattern. In general, this region is characterized by a tropical monsoon climate with seasonal reversal of winds and distinct seasonality in spatial distribution of precipitation. Due to which, NO_2_ concentrations over Bay of Bengal are observed to be low during July-Aug, 2012 (Fig. 1b) than Jan-Feb, 2012 (Fig. 1a). During June –September, winds flow from the Southern Hemisphere (SH) toward the Indian subcontinent as a southwesterly flow (Fig. 1b) accumulating moisture and depositing copious amounts of precipitation over India and the surrounding regions. Whereas the regional flow from December-February moves from Northern Hemisphere (NH) to SH as northeasterly (Fig. 1a). The shipping lane along 5–6^o^N is identified by the region having five-times of NO_2_ concentrations, of about 10 × 10^14^ molecules/cm^2^, in comparison to the surrounding region where values are around 2 × 10^14^ molecules/cm^2^. NO_2_ concentrations along the polluted shipping lane over BoB have increased at a rate of (0.08 ± 0.04) × 10^14^ molecules/cm^2^/year since 1996 (see Supplementary Fig. S2). As NO_2_ absorbs solar radiation in the spectral range 300–650 nm, it is estimated to produce an instantaneous net atmospheric heating of 2–4 Wm^−2^ and dimming (i.e., reduction of surface reaching solar radiation) of 1 Wm^−2^ in heavily polluted shipping corridors^3^,^4^. Since there are no oceanic sources of NO_2_ over this region, the observed increase in tropospheric NO_2_ values due to seagoing ships over BoB and associated warming is significantly high.

In addition to gaseous emissions (carbon dioxide, nitrogen oxides, carbon monoxide, sulphur dioxide, etc), ships exhaust also consist of unburned hydrocarbons and particulate matter such as black carbon (BC), sulphate and organic carbon. Marine vessels are contributing 7–9% of global diesel BC emissions in 2000 and is expected to rise due to increased shipping demand^5^,^6^. BC emissions further enhance the warming, as the fossil-fuel dominated black-carbon plumes are known to be more efficient warming agents^7^,^8^. The BoB, despite being a potentially very energetic region for the development of monsoon depressions, have experienced a noticeable decrease in their frequency during the recent years^9^,^10^. Also, the summer monsoon precipitation averaged over the Indian region^10^ declined by 7% during 1951–2005. Recent studies have attributed the observed declining trend of summer monsoon to anthropogenic aerosol forcing^11^,^12^,^13^,^14^. There are only few measurement studies over the BoB on the regional impact of ship emissions. The Continental Tropical Convergence Zone (CTCZ) experiment conducted during summer 2012 provided us with observations over southern BoB for determining the impact of ship emissions on black carbon warming and cloud condensation nuclei (CCN) concentration. This campaign was in part motivated by the Bay of Bengal Monsoon Experiment^15^ in 1999 and the Arabian Sea Monsoon Experiment^16^ in 2003 to broaden our understanding of monsoon processes.

## Results

**Figure 1:**
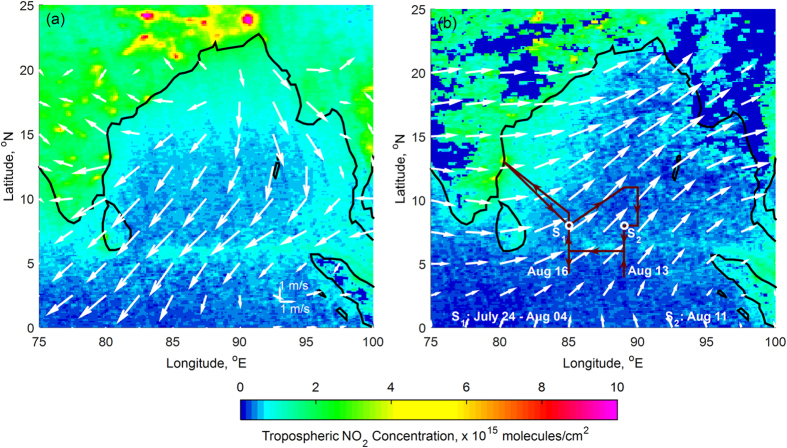
NO_2_ signature of shipping emissions in the Bay of Bengal. Mean tropospheric NO_2_ retrieved by OMI (ozone monitoring instrument) satellite for **(a)** Jan-Feb, 2012 and **(b)** July-Aug, 2012. Prevailing mean atmospheric circulation at 925 mb level during the respective time periods based on NCEP/NCAR reanalysis streamlines data provided by the NOAA-CIRES Climate Diagnostic Center are shown in white color. In (**b**) the CTCZ cruise track over southern Bay of Bengal is shown in red color. S_1_ and S_2_ shown in (**b**) are the locations where ship was stationary for continuous measurements at 8^o^N 85^o^E and 8^o^N 89^o^E respectively. Figure is generated using MATLAB R2015a software available at http://in.mathworks.com/products/matlab/. (License no: 927142).

On-board the research vessel *Sagar Nidhi*, the CTCZ campaign had deployed ground-based measurements during July 21 to August 20, 2012, over the southern BoB directly down-wind of shipping lane as shown in Fig. 1b (see Methods section). CTCZ intercepted aerosol plumes from coastal, shipping, and relatively-pristine marine sources, which were classified into three categories based on NO_2_ concentrations (Fig. 1) and the regional flow: (1) Coastal plumes, (2) Shipping plumes and (3) relatively-pristine Marine plumes, which consisted of marine sources and plumes from other regions.

### BC concentrations and associated solar heating rates

**Figure 2:**
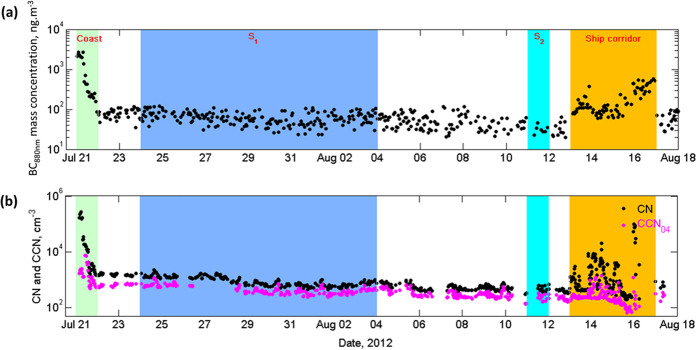
Temporal variation of measured atmospheric aerosol properties along the cruise track. **(a)** Hourly averages of black carbon (BC) mass concentration in ng.m^−3^ measured at 880 nm and **(b)** 5-min averages of total particle concentration (CN) and cloud condensation nuclei (CCN at 0.4 supersaturation) concentrations in cm^−3^. In figure, coast, ship corridor, S1 and S2 represent the data collected over coastal region, shipping corridor, 8^o^N 85^o^E (Station 1) and 8^o^N 89^o^E (Station 2) respectively.

The temporal variation of BC (at 880 nm), aerosol concentration (CN) and cloud condensation nuclei (CCN) concentrations along the cruise track are shown in Fig. 2. The BC mass concentrations were typically in the range of 30 to 2500 ng.m^−3^, with higher BCs (>200 ng.m^−3^) for anthropogenic plumes. Aerosol concentrations decreased rapidly (BC: 200 ng.m^−3^, CN: 10,420 cm^−3^) as the vessel moved 100 km away from the coast (BC: 2500 ng.m^−3^, CN: 135,490 cm) which suggested that the heavy anthropogenic emission-laden air masses encountered at the coast were transported over to the BoB. The BC (and CN) concentrations further decreased to very low values at 8^o^N (BC: 63 ng.m^−3^, CN: 775 cm^−3^), comparable to those reported for background marine concentrations of 140 ng.m^−3^ measured in the Southern Ocean^17^. We intercepted the shipping corridor at 89^o^E and 85^o^E longitudes (on Aug 12–13 and Aug 16 respectively). Owing to this, BC mass concentrations went up to 523 ng.m^−3^ with a mean value of 282 ± 184 ng.m^−3^; showing a four-fold increase in mean BC concentrations from the mean background values. In addition, we sailed zonally along 6^o^N (from 89^o^E to 85^o^E) parallel to the shipping corridor (from Aug 14–15, 2012), which allowed us to sample very short (no more than a few hours) aged aerosols from ship emissions. The mean BC concentrations at this location were 197 ± 126 ng.m^−3^. The mean BC concentration for Coastal, Shipping and relatively-pristine Marine plumes is 1,744 ± 854, 250 ± 152 and 63 ± 22 ng.m^−3^ respectively (see Table 1). Thus, commercial shipping over southern BoB had enhanced the BC concentrations along the shipping lane by a factor four and created a north-south gradient in BC concentrations. As the fossil-fuel dominated BC plumes are known to be more efficient warming agents^7^, the high BC concentrations measured over this location can influence the tropospheric temperature structure over this region.

**Figure 3:**
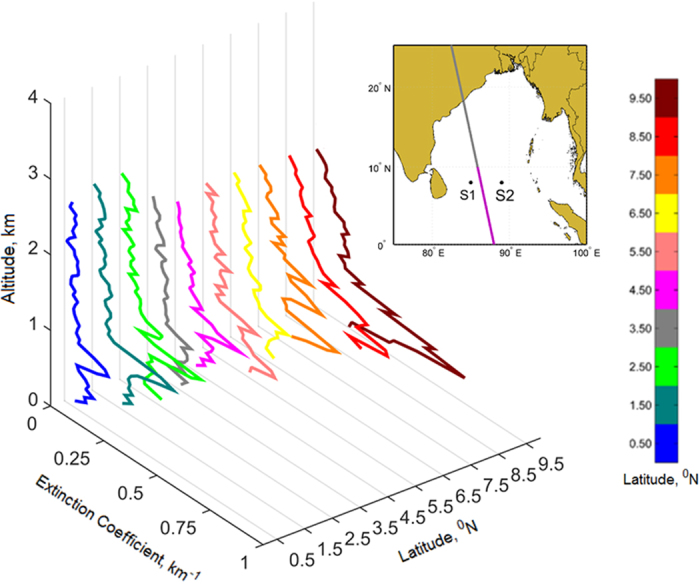
Retrieved aerosol extinction coefficient profiles obtained from CALIPSO overpass (shown in purple in the inset panel), from equator to 10^o^N along 88–89^o^E, on July 29, 2012. Extinction coefficient values were averaged over 1^o^ latitude and plotted at the middle of the latitude. This study used CALIPSO Level-2 products of aerosols with a horizontal resolution of 5 km. Figure is generated using MATLAB R2015a software available at http://in.mathworks.com/products/matlab/. (License no: 927142).

The CALIPSO (Cloud Aerosol Lidar and Infrared Pathfinder) satellite measured vertical profiles of aerosol extinction coefficient (at 532 nm) from the equator to 10^o^N on July 29, 2012, are shown in Fig. 3. July 29, 2012, data was shown as this was the only relatively-clear sky (cloudless) day where satellite overpass covered our measurement area. Moreover, clear-sky data yield the most reliable solar heating rates. Aerosol extinction coefficient, a measure of scattering and absorption of aerosols, show an increase in the atmospheric mixed layer over the shipping lane (i.e., 5.5^o^N). Extinction coefficient values south of the shipping lane were near-zero as this place experiences air-masses of marine origin (Fig. 1b); whereas values north of shipping lane were relatively high (>0.2 km^−1^) as the low-level flow brings polluted shipping emissions from shipping lane. The measured visible aerosol optical depths, AOD, a good index for column-integrated scattering and absorption, were 0.05, 0.2 and 0.1 at 4^o^N, over the shipping lane and at 8^o^N respectively.

**Figure 4:**
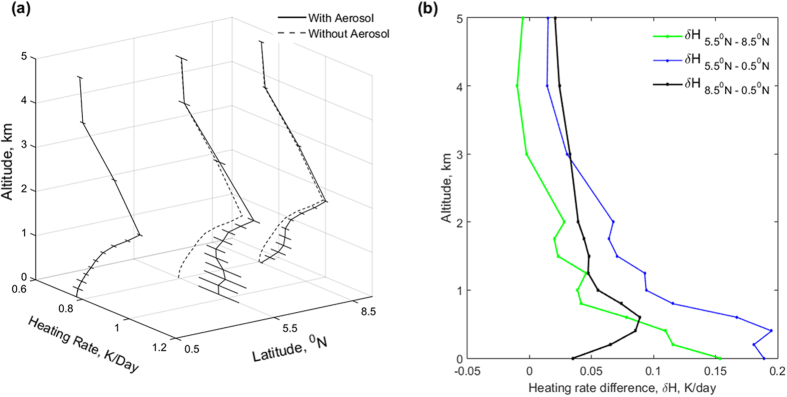
(**a**) Broadband (0.3–4.0 μm) diurnal averaged solar heating rate (H) profiles for the plumes over near-equator, shipping lane (5.5^o^N) and relatively-clean marine (8.5^o^N) for ‘aerosol’ and ‘aerosol-free’ atmospheric conditions. Horizontal bars represent the uncertainties in the estimated heating rate. (**b**) Difference in solar heating rates (δH) profiles between 5.5^o^N and 0.5^o^N (blue), 5.5^o^N and 8.5^o^N (green) and 8.5^o^N and 0.5^o^N (black).

The estimated broadband (0.3–4.0 μm) diurnal mean solar heating rates with the presence of aerosols, H, (Fig. 4a) at 1 km altitude for the ‘near-equator (0.5^o^N)’, ‘Shipping lane (5.5^o^N)’ and the ‘relatively-clean marine (8.5^o^N)’ plume conditions are respectively 0.86, 0.95 and 0.91 K/day; whereas the H within the mixed layer (~0.5 km) are respectively 0.8, 0.98 and 0.89 K/day. The enhancement in H (i.e., δH) over ‘shipping lane’ with respect to ‘near-equator’ and ‘relatively-clean marine’ region at 0.5 km altitude are about 0.18 and 0.09 K/day respectively (Fig. 4b). The aerosol-free H and aerosol H (Fig. 4a and see Supplementary Fig. S3) also confirms that the enhancement in H is due to BC, where BC heating rates over ‘shipping lane’ and ‘relatively-clean marine’ region at 0.5 km altitude are about 0.16 and 0.06 K/day respectively. In this approach also, the BC heating rate difference (ΔH) confirms to be 0.1 K/day at 0.5 km altitude between ‘shipping corridor’ and ‘marine’ location. Another confirmation for the large enhancement of heating by BC is provided by the H for the South Asian plume^18^,^19^ over the Northern Indian Ocean and East Asian plumes^7^ over Yellow Sea. For the 0.5–3 km layer, the South Asian data revealed an increase of solar heating of about 0.6[ ± 0.15]*10^−3 ^K/day per [μg/m^2^] of BC and East Asian data revealed an increase of BC heating value of about 0.5[ ± 0.2]*10^−3^ K/day per [μg/m^2^]. The vertical profile of BC concentrations over shipping corridor is obtained using satellite data (see Methods section). Integrated BC concentrations between 0.5–3.0 km altitudes over polluted shipping corridor location is 161 ug.m^−2^. The columnar BC concentrations, multiplied with diurnal heating rate per unit BC, yields diurnal mean solar-heating rate (see Supplementary Fig. S4). We derived the BC atmospheric heating rates over southern BoB for polluted shipping location to be 0.1 K/day. The close agreement validates the consistency and the statistical significance of the inferred BC heating rates.

### CCN concentrations and behavior of CCN efficiency

To estimate the impact of shipping emissions on CCN concentrations, temporal variation of CCN at 0.4% supersaturation (CCN_0.4_) measured under the varieties of conditions ranging from relatively pristine to heavily polluted conditions are shown in Fig. 2b. In general, CCN_0.4_ variations were similar to those observed for BC and CN concentrations. The CCN_0.4_ concentrations varied from 191 cm^−3^ over relatively-clean marine location to 1400 cm^−3^ over the polluted regions. The low CCN concentrations measured over relatively-clean marine locations over the BoB were comparable to those measured in clean marine conditions over the remote Atlantic^20^, Pacific Ocean^21^ and south Indian Ocean^22^. However, temporal distributions show the presence of high concentrations of anthropogenic CCN as the air parcels get impacted by anthropogenic emissions. Table 1 provides a summary of the ranges of observed aerosol measurements during each episode. The table shows a clear contrast between relatively-clean marine and anthropogenically impacted CCN concentrations which clearly demonstrate the influence of commercial ships in increasing the absorbing aerosols and CCN concentrations in a clean marine environment.

**Figure 5:**
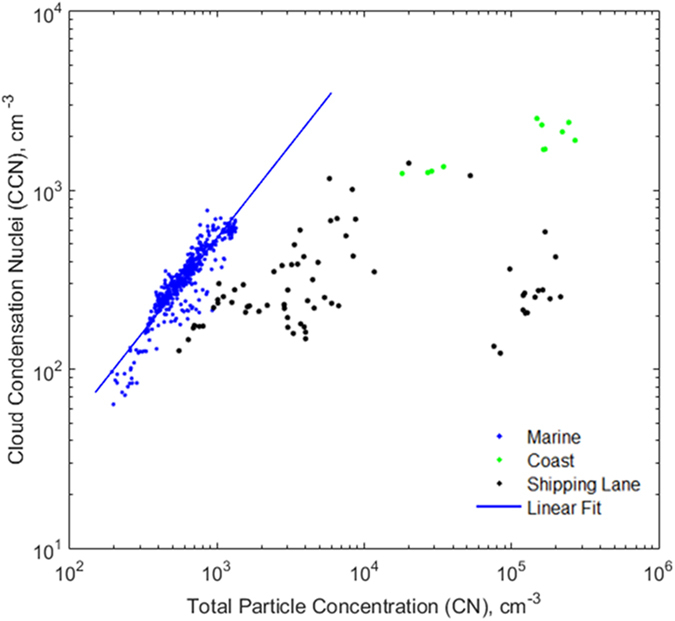
Measured aerosol concentration (CN) versus cloud condensation nuclei (CCN at 0.4 supersaturation). The blue, black and green colored data points represent marine, shipping and coastal plume conditions respectively. The continuous line is best-fit line for marine plumes, whose slope is 0.5 ± 0.02 (for 95% confidence interval).

To further determine the ability of aerosols to act as CCN at water vapour supersaturations, the relationship between CCN_0.4_ and CN concentrations are shown in Fig. 5. For the maritime air masses, the slope is close to 0.5 ± 0.02 (for 95% confidence interval) indicating that nearly all the aerosols are good CCN. The deviation of the individual points from this trend is due to somewhat less efficient CCN from anthropogenic sources. The age of these particles must be ranging from hours (given the abundance of ships in the region) to 1–2 days (the transit time). For anthropogenic air masses the scatter is much larger than for the maritime air masses, illustrating the range of tendencies to become CCN and hence, the diversity of aerosol characteristics among the air masses sampled. CCN_0.4_/CN ratio, further referred to as CCN efficiency, for a relatively-clean marine air mass was in the range 0.42 to 0.79 (see Table 1). These results are fairly consistent with other Oceanic regions, where CCN efficiencies (at 0.4% supersaturation) for pure marine cases over South Pacific^23^ was 0.33, North Atlantic^24^ was 0.41, tropical South Pacific^25^ was 0.69, tropical South Indian Ocean^26^ was 0.42 and Arctic Ocean^27^ was 0.46. The CCN efficiencies ranged from 0.0012 to 0.57 close to the shipping route due to anthropogenic activity. An air mass of ships origin would contain a large percentage of anthropogenic aerosol particles such as carbon, which is insoluble, so the relationship between CN and CCN concentration for this air mass differed from one originated over maritime.

The seaborne trade in South Asia grew by an average of 5–6% per year in recent times^28^. Regulations have already been implemented^29^ for sulphur dioxide and NO_2_ emissions and in particular efforts have been made to cut down sulphur emissions from ships^28^,^30^. The data presented in this study strongly suggest that such reductions should also be accompanied by larger percentage reductions in BC as well. Since there are no oceanic sources of BC over this region, emissions of BC from commercial ships over southern Bay of Bengal directly heat the lower troposphere by two-and-half times and increase the CCN by one order of magnitude.

## Discussion

Until now, very little work has been done on categorizing CCN concentrations over BoB during the summer monsoon season. This study allowed us to document the CCN concentration and BC warming influenced by ship emissions. A gradient of aerosol concentrations were encountered at the east coast of India and at heavily-travelled international shipping trade route. The mean BC mass concentrations varied from 63 ± 22 ng.m^−3^ (CN = 772 ± 348 cm^−3^) over relatively pristine locations to 250 ± 152 ng.m^−3^ (CN = 14,872 ± 42,300 cm^−3^) over highly polluted commercial shipping route. Seaborne trade along 5–6^o^N is a persistent anthropogenic source for BC emissions, which enhances the lower tropospheric solar heating rate by 0.16 K/day, and thus should have had a large impact on temperature profile, inversion, horizontal temperature gradient and clouds^30^,^31^,^32^,^33^.

The contrast between relatively pristine and anthropogenic aerosols over southern BoB allowed us to examine the extent to which these aerosols impact the CCN activity of aerosol. CCN concentrations over polluted shipping regions are about one-order of magnitude greater than over their remote counter parts while the BC values over the polluted region are about four-times those over their remote equivalent. CN and CCN concentrations revealed a clear linear fit for relatively-pristine air-masses, whereas the concentrations varied distinctly depending upon the extent of anthropogenic aerosols. CCN efficiency varied from 0.55 ± 0.06 over relatively pristine location to 0.29 ± 0.19 over polluted shipping corridor. In remote marine regions^34^, CCN_0.4_ are around 110 cm^−3^; whereas the mean CCN_0.4_ concentrations over relatively-pristine locations of southern BoB are 418 ± 161 cm^−3^. The enhanced CCN resulting from increase of anthropogenic aerosol emissions have the potential to disrupt organized convection in the monsoon depressions^14^. This study has shown that maritime ships in the Bay of Bengal already have a significant influence on the net warming and CCN concentrations. The myriad non-linear effects resulting from anthropogenic emissions from commercial ships over southern Bay of Bengal is an area worthy of study and regulations on ship emissions could help to reduce this impact.

## Methods

### On-board measurements

Atmospheric aerosol measurements were carried out over the southern Bay of Bengal aboard the research vessel *Sagar Nidhi* during July 21 to August 20, 2012, as part of the CTCZ (Continental Tropical Convergence Zone) campaign. The research vessel sailed from Chennai (13^o^N, 80.30^o^E) on July 21 and was back in Chennai on Aug 20 (Fig. 1b). The vessel was stationary at 8^o^N 85^o^E from July 24–Aug 4 to carry out continuous high-resolution temporal measurements (at this location vessel moved ~4 nautical miles in E-W-N-S directions for oceanic profiling only during the night-time). The vessel was stationary at 8^o^N 89^o^E for 24 hrs (on Aug 11) to carry out the inter-comparison with RAMA mooring buoy. Moreover, the vessel made continuous stoppages along the cruise track for about 40 min for ocean related measurements (station, henceforth). Data was collected at the stations as well as along the track for spatial coverage. At stations, the research vessel was manoeuvred to be heading directly upwind to avoid contamination from the ship exhaust. Nevertheless, the team encountered several contamination periods, and were recognized during the data collection period based on the wind direction, ships heading and aerosol measurements with fast time response. Such periods were isolated. All aerosol instruments were housed (approximately 15 m above the mean sea level) in a room located on the deck level in front of the vessel. Ambient air was sampled through 3/4″ stainless-steel tubing (~4 m above the deck level) and delivered to all aerosol measurement instruments through a manifold connected to the end of the sampling tube. To avoid sample losses, all tubing connecting instrumentation were routed with conductive tubing and minimal sharp bends. The aerosol concentrations (CN) were measured with a general purpose water-based condensation particle counter (GP-WCPC, Model 3787, TSI) at 1-sec time resolution. This instrument measures all particles larger than 5 nm and the reported uncertainty in CN concentrations is less than ± 10% at 250,000 particles/cm. The cloud condensation nuclei (CCN) counter (Droplet Measurement Technologies) was used to measure CCN concentrations^35^. The CCN data were obtained by changing the supersaturations about 5 min in 6 intervals (0.1%, 0.2%, 0.3%, 0.4%, 0.6% and 1.0%). The uncertainty associated with the CCN number concentrations^35^ is less than ± 10%. The black carbon (BC) mass concentrations were measured at 7-wavelengths (i.e., 370, 470, 520, 590, 660, 880 and 950 nm) using an Aethalometer (AE-31, Magee Scientific) with a time resolution of 5 min. The Aethalometer data was corrected following the procedures documented in the literature^36^. In addition, the Aerosol optical depths (AOD) were measured using Microtops instrument.

### Satellite Measurements

Monthly mean tropospheric NO_2_ concentrations were taken from satellite based instruments GOME (Global Ozone Monitoring Experiment), SCIAMACHY (SCanning Imaging Absorption spectroMeter for Atmospheric CartograpHY) and OMI (Ozone Monitoring Instrument) for the period April 1996–June 2003, July 2003–March 2012 and April 2012–December 2014 respectively^37^,^38^. The tropospheric NO_2_ columns were retrieved from the satellite observations by DOAS (Differential Optical Absorption Spectroscopy). We have employed level 3 gridded data of resolution 0.25^o^ latitude by 0.25^o^ longitudes. Combining the data from the three sensors we have formed a 19-year inventory (1996–2014) of tropospheric NO_2_ emissions over southern Bay of Bengal.

Aerosol Absorption Optical Depth (AAOD) values were derived from OMI multi-wavelength aerosol product – OMAERO (v003). It uses 17 wavelengths in the range of 330–350 nm to retrieve aerosol optical depth (AOD) and single scattering albedo (SSA) for cloud-free pixels at selected wavelengths between 330 and 500 nm. This multi-wavelength algorithm is the primary OMI retrieval method over water surfaces as it takes into advantage their low albedo in the 330–500 nm^39^. AAOD was calculated by multiplying AOD with (1–SSA). The absorption coefficient profile was obtained by scaling the aerosol extinction coefficient profile (from CALIPSO) with AAOD values (from OMI). The black carbon (BC) mass concentrations were then retrieved from aerosol absorption coefficient values.

The Cloud-Aerosol Lidar and Infrared Pathfinder Satellite Observation (CALIPSO)^40^ is part of the “A-Train” constellation consisting of three other satellites–Aqua, Aura and CloudSat. CALIPSO uses an active lidar instrument along with passive infrared and visible imagers to probe the vertical structure and properties of thin clouds and aerosols. The extinction coefficients considered in this study are level 2, Lidar Aerosol Layer Data from CALIOP (Cloud-Aerosol LIdar with Orthogonal Polarization) instrument on-board CALIPSO.

### Model Calculations

Atmospheric heating rates are estimated by inputting the satellite retrieved aerosol vertical profile into the MODTRAN (short for MODerate resolution radiative TRANsfer program) code^41^. The clear-sky (i.e., cloud-free condition) calculations were performed with MODTRAN5 code, which is the latest version of its radiative transfer code. The code simulates fluxes at mid-ultraviolet to visible to far-infrared bands to cover the solar spectrum from 0.2 to 100.0 μm. Broadband integration in the shortwave region (0.2–4.0 μm) was carried out to estimate fluxes (incoming and reflected), atmospheric solar absorption and heating rates at multiple altitudes. Correlated k-distributions were used to incorporate gaseous absorption by water vapour, ozone, oxygen, carbon dioxide etc. (see MODTRAN manual for more details^41^). The model accounts for all kinds of multiple scattering and absorption by individual aerosol species, cloud droplets, air molecules and reflections from the surface. The underlying surface was considered to be oceanic surface. The oceanic surface albedos incorporated in the code were calculated according to *Briegleb et al.*^42^. Observed aerosol properties collected at the surface (including sun photometer data) and satellite-retrieved aerosol extinction coefficient and absorption coefficient vertical profiles were used as input parameters for the calculations. Aerosol extinction coefficient profiles were obtained by scaling the satellite retrieved extinction coefficient profiles with measured AOD data. Measured vertical profiles of pressure, temperature and water vapour concentration from the CTCZ experiment were used in the calculations. The instantaneous fluxes were calculated and converted to diurnal mean values using the MODTRAN radiative transfer model.

## Additional Information

**How to cite this article**: Ramana, M. V. and Devi, A. CCN concentrations and BC warming influenced by maritime ship emitted aerosol plumes over southern Bay of Bengal. *Sci. Rep.* **6**, 30416; doi: 10.1038/srep30416 (2016).

## Supplementary Material

Supplementary Information

## Figures and Tables

**Table 1:** Summary of parameters during the selected episodes.

**Aerosol episodes(→)**	Coastal Range(Mean ± **SD)**	Relatively clean marineRange (Mean ± **SD)**	Shipping Lane Range(Mean ± **SD)**
BC_880 nm_ (ng.m^−3^)	420–2,662 (1,744 ± 854)	30–142 (63 ± 22)	97–548 (250 ± 152)
CN (cm^−3^)	18,110–2,70,652(1,35,486 ± 93,675)	350–1,851 (772 ± 348)	193–2,15,814(14,872 ± 42,300)
CCN_0.4_ (cm^−3^)	1,245–2,525 (1,801 ± 486)	191–938 (418 ± 161)	64–1,420 (291 ± 209)
CCN_0.4_/CN	0.01–0.07 (0.03 ± 0.02)	0.42–0.79 (0.56 ± 0.06)	0.0012–0.57 (0.29 ± 0.19)

[Coastal plumes: July 21, 2012 (coast to 100 km); Relative clean marine: July 23–Aug 11, 2012; shipping plumes: Aug 13–16, 2012].
