# Supplementary material for: CCN concentrations and BC warming influenced by maritime ship emitted aerosol plumes over southern Bay of Bengal
Source: Sci Rep. 2016 Aug 2;6:30416. doi: 10.1038/srep30416 (PMC4969613; doi:10.1038/srep30416)
Supplement: Supplementary Information [file srep30416-s1.pdf]

SUPPLEMENTARY INFORMATION

CCN concentrations and BC warming influenced by maritime ship emitted aerosol plumes over southern Bay of Bengal

M V Ramana[*,ɣ] **and Archana Devi**

Indian Institute of Space Science and Technology, Thiruvananthapuram, Kerala, 695 547, India.

[ɣ] Currently at: National Remote Sensing Centre, Hyderabad, Telangana, 500 037, India.

[*]Corresponding author: ramana@iist.ac.in

Contact information:

M. V. Ramana
Associate Professor,
Indian Institute of Space Science and Technology
Thiruvananthapuram – 695 547, Kerala, India.

Phone: 91-9995424965 (mobile)
E-mail: ramana@iist.ac.in

Supplementary Figure S1

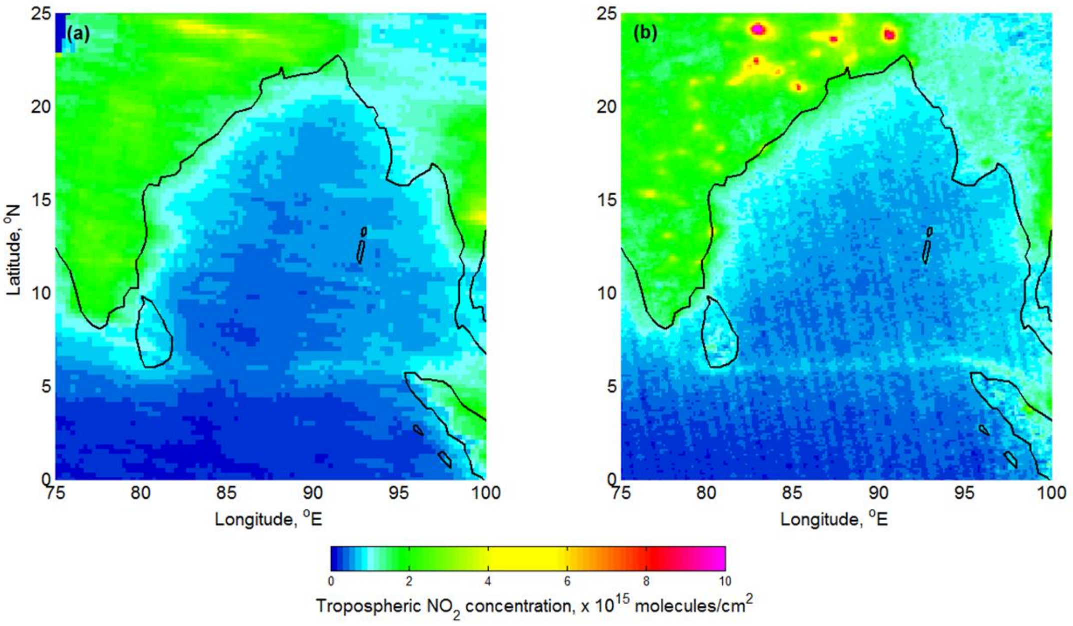

Supplementary Figure S1: Satellite measurements of annual mean tropospheric NO_2 column concentrations retrieved by **(a)** GOME for the year 1997 and **(b)** OMI for the year 2015 over the Bay of Bengal region. A discernible increase in the annual mean tropospheric NO_2 concentrations can be seen at ~5-6°N which overlaps with the shipping lane between Sri Lanka and Indonesia. Hence this increase is attributed to shipping emissions. Over the continent, fossil fuel emissions may have increased the NO_2 concentrations in major cities. Moreover, continental outflow of pollution has carried these NO_2 emission from the coasts to relatively pristine marine regions. Relatively high NO_2 concentrations over Indonesian region in Fig (a) is due to 1997 Indonesian forest fires that increased the NO_2 concentrations over Indonesia and its neighbouring regions.

The datasets used in this study are GOME (Jan 1997 - Dec 1997) and OMI (Jan 2015 - Dec 2015) monthly datasets. GOME resolution is 0.25^o x 0.25^o and OMI resolution is 0.125^o x 0.125^o. Annual mean tropospheric NO_2 column concentrations over respective girds were calculated by averaging monthly mean tropospheric NO_2 column concentrations. Figure S1 is generated using MATLAB R2015a software available at http://in.mathworks.com/products/matlab/. (License no: 927142)

Supplementary Figure S2

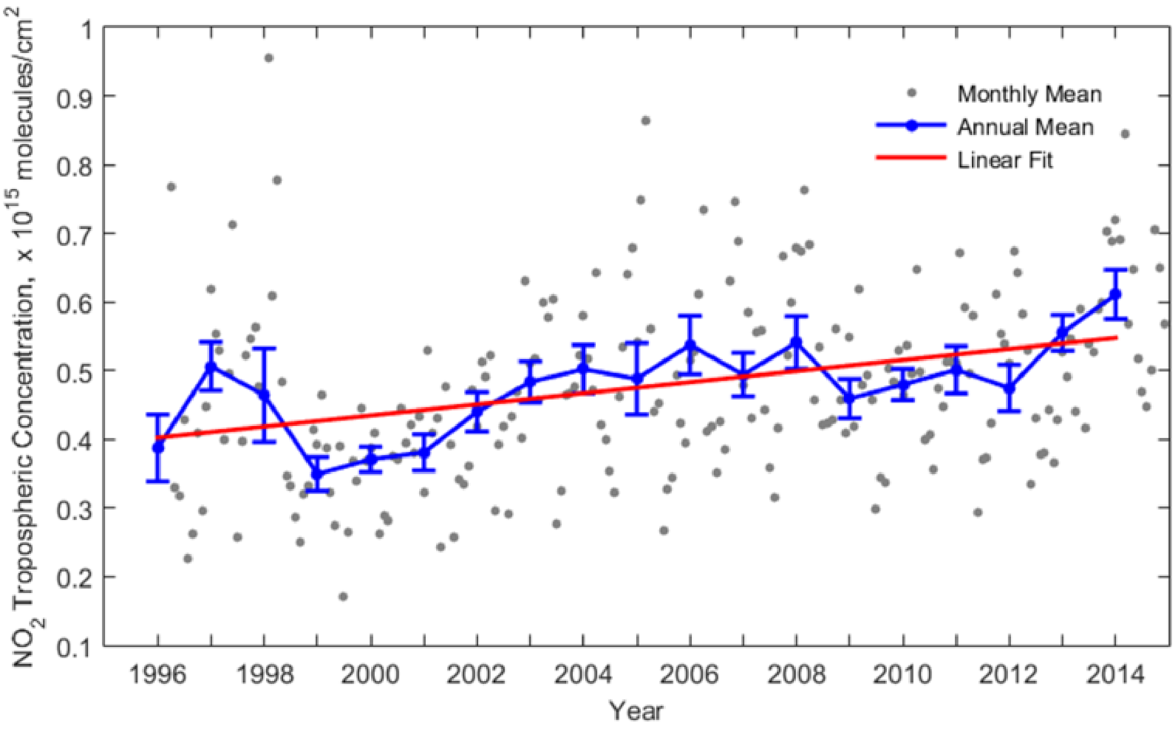

Supplementary Figure S2: 1996 - 2014 time-series of tropospheric NO_2 column concentration for the shipping lane over Bay of Bengal. Grey circles indicate monthly mean and blue circles the annual mean NO_2 tropospheric concentration with error bars representing standard error of mean. The linear fit over annual mean values, denoted by the red line, shows an increase of NO_2 concentrations at a rate of (0.08 $\pm$ 0.04) x 10^{14} molecules/cm^2/year (for 95% confidence interval).

The datasets used in this study are GOME (April 1996 - June 2003), SCIAMACHY (July 2003 – March 2012) and OMI (April 2012 – December 2014). The OMI satellite data sets have been rescaled to match the coarser resolution of GOME and SCIAMACHY data. The abrupt increase in NO_2 concentration over the shipping lane during 1997-1998 is due to the influence of 1997 Indonesian forest fires.

Supplementary Figure S3

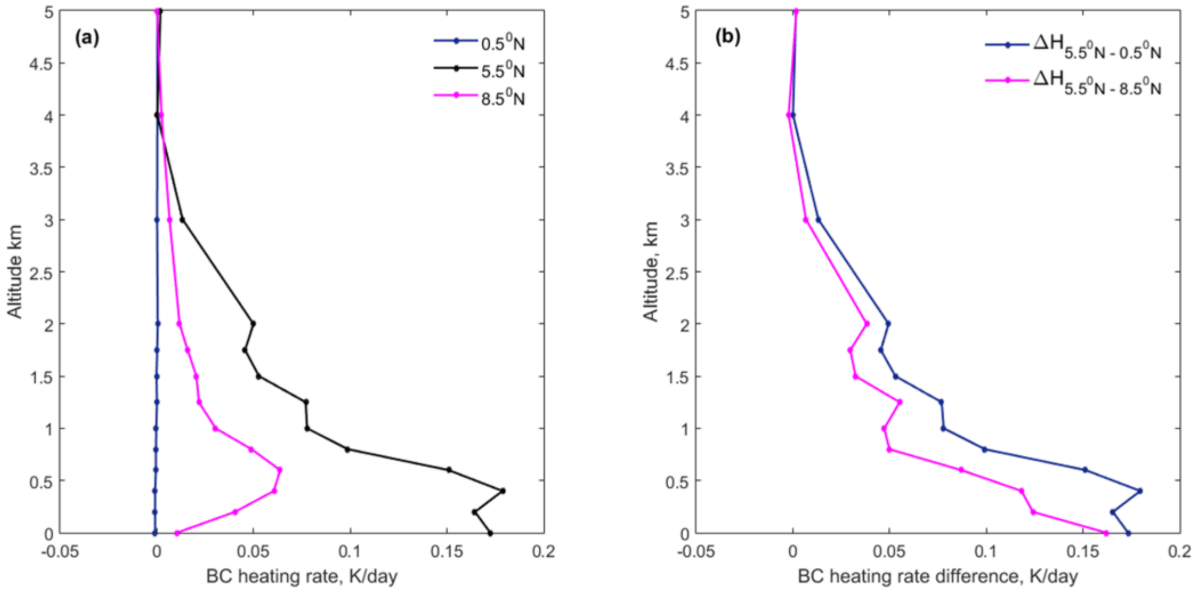

Supplementary Figure S3: (a) Black carbon (BC) heating rates at 0.5°N, 5.5°N and 8.5°N are obtained by subtracting solar heating rates for aerosol-free atmosphere from solar heating rates with aerosol (See Figure 4a). **(b)** Difference in solar heating rate (ΔH) profiles between 5.5°N and 0.5°N (blue) and 5.5°N and 8.5°N (pink) to understand the heating rate gradient.

Supplementary Figure S4

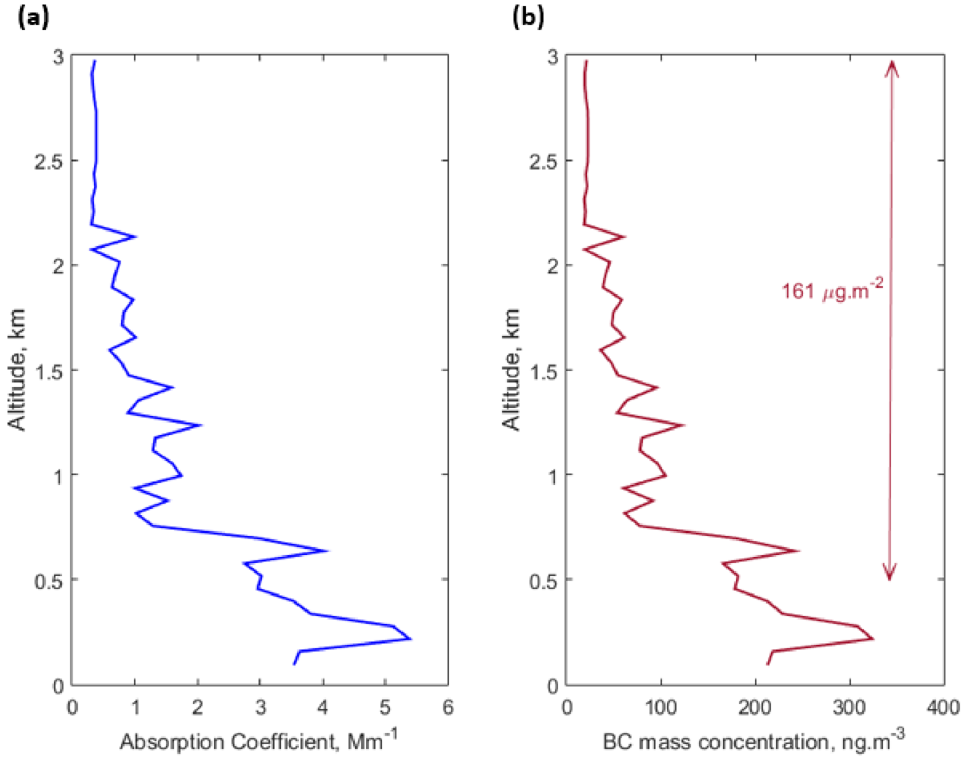

Supplementary Figure S4: Vertical profiles of **(a)** aerosol absorption coefficient (Mm^{-1}) and **(b)** black carbon (BC) mass concentration (ng. m^{-3}) at 5.5^{o}N (shipping corridor location) obtained from the satellite data. Absorption aerosol optical depths (AAOD) are obtained from OMI satellite data [OMI satellite provides aerosol optical depth (AOD) and single scattering albedo (SSA) from which AAOD was calculated by multiplying (1- SSA) with AOD]. The aerosol absorption coefficient profile is obtained by scaling the aerosol extinction coefficient profile (CALIPSO data) with AAOD value. Black carbon (BC) mass concentration values are then retrieved from aerosol absorption coefficient values.

Integrated black carbon concentrations at 5.5^{o}N between 0.5-3 km altitudes is 161 µg.m^{-2}.

Diurnal mean heating rate per unit BC is: 0.6[±0.15]*10^{-3} K/day per [µg/m^2] of BC

Diurnal mean solar-heating rate = (161 µg.m^{-2})* (0.6[±0.15]*10^{-3} K/day per [µg/m^2])

= 0.097 K/day ≡ 0.1 K/day
